# Spontaneous SSCD Auto-Plugging: Clinical, Electrophysiological and Radiological Evidence

**DOI:** 10.3390/jcm14228054

**Published:** 2025-11-13

**Authors:** Pierre Reynard, Eugenia Mustea, Aïcha Ltaief-Boudrigua, Andrea Castellucci, Hung Thai-Van, Eugen C. Ionescu

**Affiliations:** 1 Department of Audiology and Otoneurological Explorations, Civil Hospitals of Lyon, 69003 Lyon, France; eugenia.mustea@chu-lyon.fr (E.M.); hung.thai-van@chu-lyon.fr (H.T.-V.); eugen.ionescu@chu-lyon.fr (E.C.I.); 2 Paris Hearing Institute, Institut Pasteur, Inserm U1120, 75015 Paris, France; 3 Medicine Department, Claude Bernard Lyon 1 University, 69003 Lyon, France; 4 Department of Radiology, Hospices Civils de Lyon, 69003 Lyon, France; aicha.ltaief-boudrigua@chu-lyon.fr; 5 ENT Unit, Department of Surgery, AUSL—IRCCS di Reggio Emilia, 42123 Reggio Emilia, Italy; 6 ENT Department, Carol Davila University of Medicine, 050474 Bucharest, Romania

**Keywords:** otic capsule dehiscence, superior semicircular canal dehiscence, superior semicircular canal dehiscence auto-plugging

## Abstract

**Background:** Superior semicircular canal dehiscence (SSCD) is characterized by a bony defect of the superior semicircular canal (SSC), leading to vestibular and auditory symptoms. A process of spontaneous “auto-plugging,” in which the overlying dura mater progressively occludes the SSC, may replicate the effects of surgical canal plugging but remains under-recognized. The present study reports diverse clinical, instrumental, and 3d High Resolution MRI findings in patients with SSCD and subsequently confirmed to present with spontaneous complete or partial auto-plugging. **Methods:** We retrospectively reviewed 11 patients with SSCD diagnosed on high-resolution CT and suspected auto-plugging based on clinical atypia and large dehiscence (>4 mm). Patients underwent comprehensive neurotological assessment, including pure-tone audiometry, vestibular testing, and HR MRI with 3D labyrinthine reconstructions to identify partial or complete auto-plugging. Auto-plugging was classified as partial (Canalis semicircularis superior depressus) or complete (absence of endolymph fluid signal; Canalis semicircularis superior obturatus). **Results:** Among 13 ears with auto-plugging, 6 were partial and 7 complete. The mean SSCD size in auto-plugged ears was 5.5 mm. Most ears had normal or near-normal vestibular function on VHIT, with minimal air-bone gaps and preserved VEMP responses. Imaging demonstrated varying degrees of dural contact with the SSC, confirming partial or complete canal occlusion. **Conclusions:** Spontaneous auto-plugging of the SSC is a plausible, under-recognized phenomenon that may reproduce functional effects of surgical plugging. Dedicated 3D labyrinthine MRI enhances detection and characterization. Prospective multimodal studies are needed to clarify the pathophysiology, progression, and clinical implications, optimizing patient selection for surgical versus conservative management.

## 1. Introduction

Cawthorne was the first to introduce the term third otic window in the context of vestibular fenestration surgery for advanced otosclerosis [1]. This concept was further supported by Tullio’s experiments, in which the creation of an additional otic window in animal models led to specific vestibular responses to loud sound and/or vibration, thereby reinforcing the hypothesis of an alternative mobile window influencing inner ear fluid dynamics [2]. Minor first described spontaneous dehiscence between the dura and the superior semicircular canal (SSC), known as superior semicircular canal dehiscence (SSCD) [3,4]. Expanding on this, Wackym et al. proposed the broader concept of otic capsule dehiscence syndrome, encompassing all third mobile windows (TMWs) abnormalities characterized by symptoms, clinical signs, and audiometric findings consistent with bony defects of the otic capsule, confirmed by high-resolution computed tomography (HRCT) [5].

The absence of bony coverage at the SSCD site increases the compliance of the exposed membranous labyrinth, facilitating perilymphatic shunting toward the dehiscent area rather than through the basilar membrane, and producing an “inner-ear conductive hearing loss” pattern [4,6,7]. Clinical manifestations include pressure- and sound-induced vertigo or hypersensitivity, hyperacusis to bone-conducted sounds, pulsatile tinnitus, and chronic imbalance [4,5,8]. While conservative medical management may suffice for patients with mild symptoms, definitive treatment for SSCD typically involves surgical intervention via middle cranial fossa and/or transmastoid approaches. These procedures aim to “plug” the SSC, thereby functionally excluding it from the vestibular circuit [9]. Surgical techniques include SSC plugging, resurfacing, or a combination of both [10].

A process of spontaneous “auto-plugging” has been proposed, in which progressive herniation of the dura mater from the middle cranial fossa into the SSC occurs through the dehiscence, ultimately leading to natural canal occlusion [4,11,12,13]. This phenomenon is thought to replicate the effects of surgical canal plugging. Cremer et al. first hypothesized this mechanism for large-sized SSCD [14]. Castellucci et al. suggested a spontaneous plug in a case of SSCD caused by a temporal bone meningocele, and Ionescu et al. subsequently confirmed it using high-resolution 3D labyrinthine MRI [15,16,17]. Furthermore, Castellucci et al. reported similar reductions in vestibulo-ocular reflex (VOR) gain for the affected canal in some patients with SSCD and in those who underwent surgical SSC plugging [18]. These findings suggest that spontaneous plugging may, in certain cases, reproduce the functional and imaging outcomes of surgical intervention, although not always accompanied by typical TMW abnormalities. Atypical patterns may include near-normal vestibular-evoked myogenic potential (VEMP) thresholds or amplitudes and minimal or absent air-bone gaps on audiometry.

Usual diagnostic workup relies exclusively on HRCT with Pöschl plane reconstruction, without the benefit of MRI with T2-weighted HR sequence on inner ear. The first case of partial spontaneous SSCD auto-plugging confirmed by 3D labyrinthine MRI was reported by Ionescu et al., in which the VOR gain of the affected canal remained normal [16]. This observation raised new questions regarding the clinical and instrumental features of SSCD cases demonstrating MRI evidence of auto-plugging. Moreover, some SSCD patients with recurrent Menière-like symptoms may experience them as a consequence of spontaneous SSC occlusion by the overlying dura, a hypothesis supported by Brandolini et al. based on the symptomatic resemblance to patients in the immediate postoperative period following SSC plugging [11].

Using HRCT of the petrous bone combined with 3D labyrinthine MRI sequences, the present single-center study reports diverse clinical, instrumental, and imaging findings in a series of patients with definite or probable SSCD, defined according to the Bárány Society criteria, and subsequently confirmed to present with spontaneous complete or partial auto-plugging.

## 2. Materials and Methods

### 2.1. Population

Between January 2021 and January 2025, a retrospective review was conducted including all consecutive patients diagnosed with SSCD on HRCT and suspected of auto-plugging according to both of the following criteria:

− Clinical atypia, defined as presentation not fully consistent with the Bárány Society’s recommended diagnostic criteria [8], characterized by one or more of the following features:
  − No or lesser than expected conductive hearing loss (CHL) in pure tone audiometry: no or slight air bone gap (ABG)
  − Normal cVEMP threshold, normal oVEMP amplitude
  − No bone conduction hyperacusis (including autophony)
  − No pulsatile tinnitus (PT)
  − No nystagmus or dizziness induced by pressure or sound
  − Arguments for another possible vestibular disease or disorder.
− And a large dehiscence in HRCT, defined here as >4 mm in the Pöschl plane (Suspicion of auto-plugging)

In all patients with both criteria, additional 3T MRI on inner ear with T2-weighted HR sequence, and 3D labyrinthine reconstructions was performed to explore potential anatomical correlates.

Exclusion criteria included: patients under 18 years of age; individuals with a personal or family history of migraine, motion sickness, other middle or inner ear malformations, central nervous system disorders, ophthalmologic or vergence abnormalities, psychological conditions, or systemic illnesses. Patients with insufficiently clear radiological data were also excluded.

This study was conducted in accordance with the ethical principles outlined in the Declaration of Helsinki (https://www.wma.net/wp-content/uploads/2016/11/DoH-Oct2013-JAMA.pdf, accessed on 23 December 2024). Written informed consent was obtained from all participants.

### 2.2. Audio-Vestibular Assessment

A standard neurotological examination, including cranial nerve evaluation and oto-microscopy, was routinely performed in all patients. Pure tone audiometry (Madsen Astera-Otometrics, Taastrup, Denmark), middle ear reflexes (Madsen Zodiac 901 tympanometer, Taastrup, Denmark), videonystagmography including bone vibratory test (BVT) and Valsalva maneuver (VNG, Ulmer^®^, Synapsis SA, Marseille, France), video head impulse test (VHIT, Ulmer^®^, Synapsis SA, Marseille, France), cervical vestibular evoked potentials (cVEMPs) and ocular vestibular evoked potentials (oVEMPS) (Bio-Logic^®^ Nav-Pro system, Mundelein, IL, USA) in air conduction with 750 Hz stimuli were systematically performed in all patients. During data acquisition, recordings were stopped once consistent VEMP responses were obtained. Threshold recordings were repeated at least twice to ensure reliability. We collected two parameters for each ear: the response amplitude (P-to-N peak amplitude in µV) after presentation of the stimulus at 95 dB nHL, and the response threshold (dB nHL), determined by progressively decreasing the stimulus intensity.

### 2.3. Radiological Assessment

All patients underwent HRCT of the temporal bone using the GE GSI Revolution scanner (GE Healthcare, Chicago, IL, USA). Axial helical acquisitions were performed with a nominal slice thickness of 0.625 mm and a 50% overlap (0.312 mm). Images were obtained in ultra-high-resolution mode at 140 kV and 200 mAs/section. Multiplanar reconstructions were performed in the Pöschl plane (aligned with the SSC) using the Advantage Workstation (AW Server, GE Healthcare, Chicago, IL, USA). The size of the SSCD was calculated by tracing the missing curved segment between the bony remnants of the SSC and measuring its length.

Additionally, 3T MRI with T2-weighted HR sequences targeting the labyrinth were obtained to evaluate suspected spontaneous membrane collapse over the SSC (MR 7700, Philips, Amsterdam, The Netherlands). Inner ear 3D imaging was performed using high-resolution T2-weighted DRIVE (Driven Equilibrium) sequences with a dedicated head coil. The sequence parameters were: isotropic voxel size 0.5 × 0.5 × 0.5 mm, TR ≈ 1650 ms, TE ≈ 187 ms, matrix 300 × 300, FOV 150 × 150 mm, and total scan time under 15 min per sequence with Compress Sens acceleration software (SmartSpeed with Compressed SENSE 2.0, MR 7700 system (software release R12.3), Philips Healthcare, Amsterdam, The Netherlands). Multiplanar reconstructions and fusion imaging were performed to assess the membranous labyrinth and semicircular canals morphology.

As our series included cases showing complete auto-plugging as well as intermediate forms—with early, moderate, or partial obstruction of the superior semicircular canal, but without complete compromise of its lumen—we propose the following classification.

-Partial auto-plugging: MRI shows deformation/beveling of the SSC apex, but some fluid remains (we use the term “Canalis semicircularis superior depressus”).

-Complete auto-plugging: MRI shows complete loss of endolymphatic signal in the SSC. We propose the term “Canalis semicircularis superior obturatus”).

## 3. Results

### 3.1. Clinical and Radiological Findings

**Figure 1:**
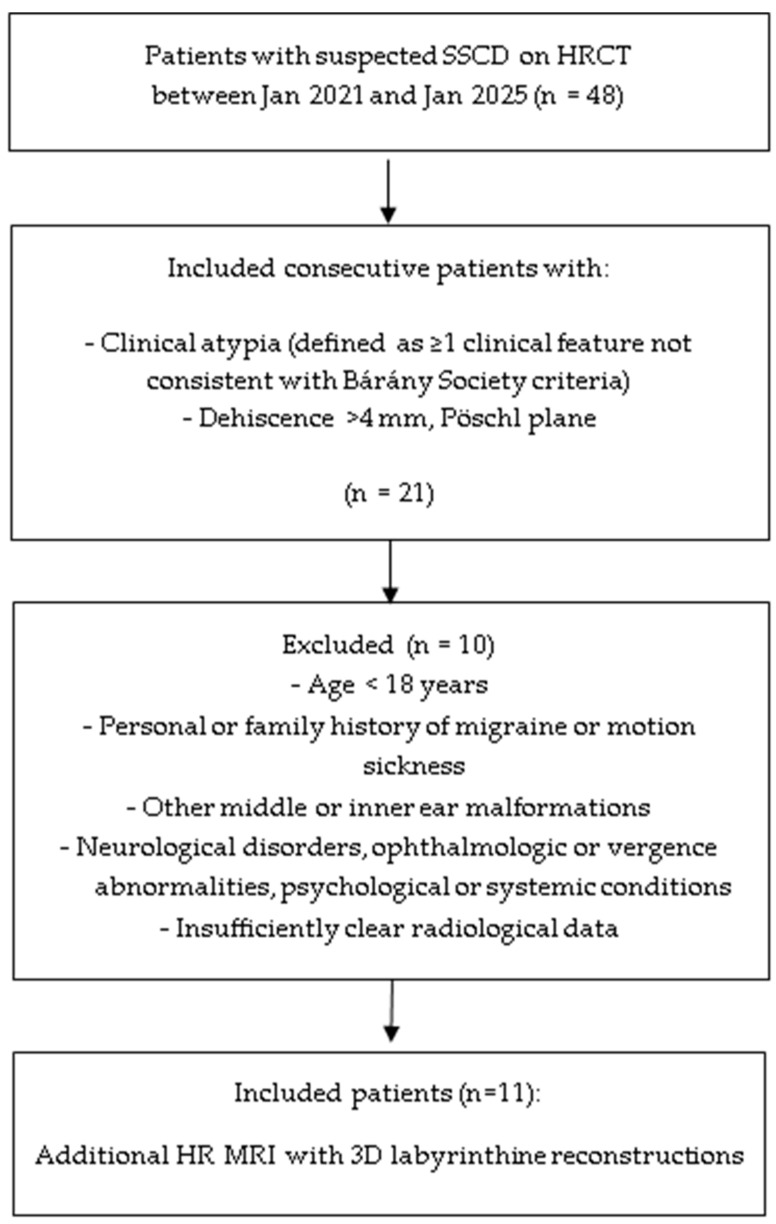
Consort 2010 flow diagram for inclusions.

A total of 11 patients were included (Figure 1); epidemiological, clinical, and instrumental data are summarized in Table 1. The sex ratio was 6 females to 5 males, and the mean patient age was 53.8 years (range: 37–85). Radiological findings for all patients are presented in Table 2. Bilateral SSCD was identified in 8 out of 11 patients. Not all SSCDs were auto-plugged; across the 11 patients, a total of 13 ears with auto-plugging were found (6 partial, 7 complete). The mean SSCD size in auto-plugged ears was 5.5 mm. The mean size was slightly higher in cases of complete auto-plugging (5.7 mm) compared to partial auto-plugging (5.3 mm).

### 3.2. Audio-Vestibular Findings

Conductive hearing loss (CHL) was present in 10 of 13 auto-plugged ears, while audiometry was normal in one ear and revealed sensorineural hearing loss (SNHL) in two (Table 2). PT was reported in four patients (on the side of the auto-plugging). Autophony was present in 5 patients (on the side of the auto-plugging). Dizziness was reported in four patients and pressure- or noise-induced vertigo in 3 patients.

cVEMPs were performed in all patients. The mean threshold for partial and complete auto-plugging was 58.3 dB HL and 59.3 dB HL, respectively. A normal threshold was found in only one ear with SSCD auto-plugging. oVEMPs were performed in 9 out of 11 patients; responses were present in all ears with SSCD auto-plugging.

VHIT gains were normal in 9 out of 11 patients; in 2 patients (3 ears with auto-plugged SSCD), we observed a slight VOR impairment of the SSC (gain = 0.6).

The phenotypic variability was remarkable, ranging from a 68-year-old female (Patient 1) with moderate mixed left hearing loss and only effort-related vertigo to an asymptomatic patient (Patient 8) with normal audiometry, in whom the dehiscence was incidentally found on MRI performed for continuous non-pulsatile left tinnitus; notably, this latter case also showed normal cVEMP thresholds, unlike the majority of other auto-plugged ears in the series.

### 3.3. Imaging Presentation

**Figure 2:**
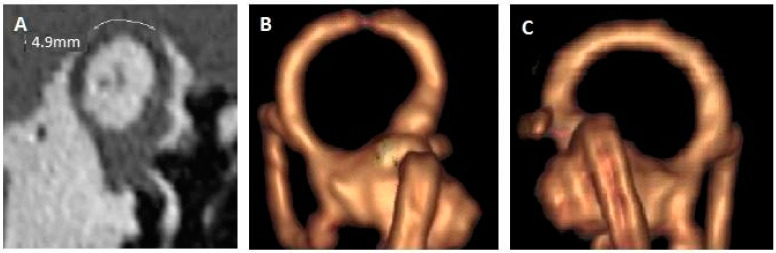
Left SSCD with partial auto-plugging. (**A**) HRCT, Pöschl plane; dehiscence measured at 4.9 mm. (**B**) 3D HR MRI, reconstruction in the plane of the SSC (left ear). (**C**) 3D HR MRI, reconstruction of the labyrinth, right ear; the image is provided for comparison. MRI: magnetic resonance imaging; HRCT: high resolution cranial tomodensitometry; SSCD: superior semicircular canal dehiscence.

**Figure 3:**
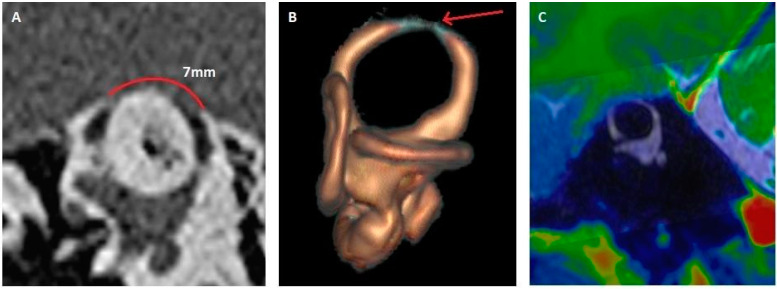
Right SSCD with complete auto-plugging. (**A**) HRCT, Pöschl plane; dehiscence measured at 7 mm. (**B**) 3D HR MRI, reconstruction in the plane of the SSC. Complete auto-plugging (arrow) (**C**) fusion between HRCT and 3D T1 MRI. MRI: magnetic resonance imaging; HRCT: high resolution cranial tomodensitometry; SSCD: superior semicircular canal dehiscence.

Figure 2 shows the 3D HR MRI appearance obtained for patient number 5. The SSCD was measured at 4.9 mm on HRCT (Figure 3A), and MRI demonstrates a partial auto-plugging on the left side, with an image of a defect at the SSC apex, but some endolymph remains (Canalis semicircularis superior depressus) (Figure 3B). On the right side, the dehiscence was small on HRCT (2 mm). On the 3D HR MRI image, there is no evidence of auto-plugging, even partial (Figure 3C). Figure 3 shows the 3D HR MRI appearance obtained for patient number 6. The right SSCD was large (7 mm); 3D HR MRI showed complete right auto-plugging with complete loss of endolymphatic signal (Canalis semicircularis superior obturatus). For this patient, fusion sequences between HRCT and 3D T1 sequence were performed to ensure that no vascular structure compressing the canal at the interface.

**Figure 4:**
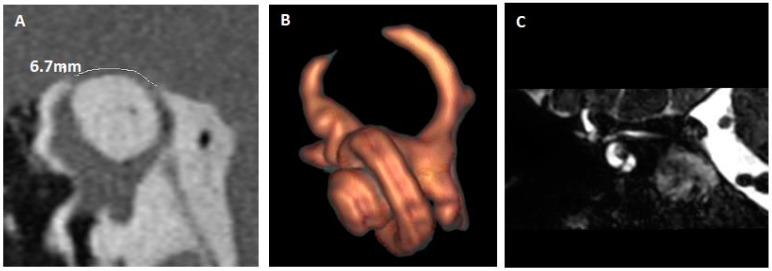
Right SSCD with complete auto-plugging. (**A**) HRCT, Pöschl plane; dehiscence measured at 6.7 mm. (**B**) 3D HR MRI, reconstruction in the plane of the SSC (right ear). (**C**) 3D T2 high-resolution MRI (DRIVen Equilibrium pulse). MRI: magnetic resonance imaging; HRCT: high resolution cranial tomodensitometry; SSCD: superior semicircular canal dehiscence.

Another example of complete auto-plugging is shown in Figure 4 (patient 7). The coronal T2 DRIVen equilibrium pulse MRI sequence demonstrates the dura mater very close to the right SSC, suggesting an auto-plugging, which was confirmed on reconstruction.

The full collection of patient images is available in the Supplementary Materials. It should be noted for 3D HR MRI reconstructions that the image rendering may vary between patients due to different signal recovery thresholds. The orange appearance is related to the fact that the threshold for recovering some signal was broadened (particularly for the lateral semicircular canal, which is affected by artifacts; a defect is typically seen on 3D reconstructions due to the close interface between bone and air) compared to the more or less white aspect.

## 4. Discussion

The principal finding of this study is the MRI-based characterization of SSCDs, highlighting the presence of partial or complete spontaneous auto-plugging. Nevertheless, several clinical and instrumental aspects warrant further consideration. All patients exhibited incomplete or atypical symptom profiles of TMW. It is well established that a subset of SSCD patients may lack classical features such as CHL; therefore, MRI evaluation for auto-plugging should be considered a complementary step following HRCT identification of a dehiscence, as this may account for the heterogeneity of clinical presentations [16,17,19,20,21]. No consistent clinical or instrumental distinctions were identified between partial and complete plugging. In all patients with auto-plugging, cVEMPs demonstrated lowered thresholds, consistent with typical SSCD findings [8]. Importantly, auto-plugging did not result in threshold normalization, in contrast to the normalization frequently reported after surgical canal plugging [20].

Unexpectedly, SSC VHIT deficits were identified in only two patients. Carey et al. reported preoperative VOR gain reductions (0.7, n = 19) in SSCD patients using the magnetic search coil technique, with a further postoperative decrease of approximately 40% [12]. Mukherjee et al. proposed that isolated SSC VHIT abnormalities may be characteristic of SSCD [13], while Castellucci et al. documented VOR gain reductions both in SSCD patients and in those who underwent surgical SSC plugging, suggesting that spontaneous auto-plugging could account for these similarities [18]. In our cohort, the observation of SSC impairment at high head velocities in only two patients could indicate that auto-plugging may normalize the VOR response. However, the limited sample size prevents definitive conclusions, and normal VOR gain was also found in our study in SSCD ears without imaging evidence of auto-plugging.

As suggested by Minor and later by Castellucci et al., a reduction in VOR gain may result from endolymphatic flow dissipation through the dehiscent site during rapid head impulses, independent of any plugging process [4,18,19]. In this model, diminished fluid-mechanical stimulation of the SSC cupula during high-velocity movements produces an apparent hypofunction, often described as “pseudo-hypofunction.” Similar results have also been reported in cases with posterior semi-circular canal dehiscence, where the impaired VOR gain value for the affected canal has been attributed to a dispersion of endolymphatic flow energy rather than to a spontaneous auto-plugging, which seems less likely to occur in that position [22]. In fact, due to the integrity of the bony labyrinthine, it is assumed that the perilymphatic compartment contributes to cupular activation through mechanical deformation of the membranous ducts, supporting and complementing the deflection mechanism driven by cupular and endolymphatic dynamics [7]. In this context, auto-plugging may reinforce the perilymphatic space, which is otherwise unsupported in SSCD, thereby improving endolymphatic flow toward the cupula during high-frequency head impulses and explaining the preservation of normal high-velocity VOR gain values.

A relationship between SSCD size and cVEMP amplitude, cVEMP threshold, as well as air-conduction audiometric thresholds or the air–bone gap has already been described [23,24]. With respect to VOR gain reduction on VHIT in correlation with SSCD size, Castellucci et al. further hypothesized the involvement of an auto-plugging mechanism [24]. Similarly to Cremer et al. [14], we propose that partial or complete auto-plugging is more likely to occur in the presence of a large SSCD. However, in our series we did not observe a systematic reduction in VOR gain among the included patients. One possible explanation is that the previously reported correlation holds true only up to a certain threshold, after which the onset of auto-plugging may lead to a subsequent trend toward normalization of audiometric thresholds, VOR, and cVEMPs. Preserved SSC function has occasionally been reported after surgical plugging [25,26], likely reflecting residual cupular deflections mediated by endolymphatic redistribution in the non-occluded canal segment. Central compensatory mechanisms may further account for normal VOR gain, particularly in cases of slowly progressive auto-plugging. Whether partial auto-plugging results in greater symptom burden or distinct instrumental findings compared with complete auto-plugging remains unresolved. Notably, after surgical plugging of a symptomatic superior or posterior canal, the closer the occlusion is to the cupula, the more consistently VOR abolition is observed for the involved canal [25].

### 4.1. Radiological Elements

Although HRCT remains the anatomical gold standard for evaluating bony dehiscence [8], modern 3D T2-weighted MR sequences (e.g., constructive interference in steady state (CISS), DRIVEn equilibrium, Sampling Perfection with Application optimized Contrast using different flip angle Evolution (SPACE)) reconstructed in the Pöschl plane demonstrate strong diagnostic concordance and, importantly, offer unique postoperative and natural history insights beyond the capability of HRCT [16,17]. These include direct visualization of canal lumen occlusion, membranous collapse, or incomplete surgical plugging [27,28,29]. Such MRI findings are consistent with the auto-plugging hypothesis and may help explain apparently discordant physiological results, such as near-normal VHIT gains despite functional canal isolation [16,17,29]. MRI has now documented both partial and, more recently, complete spontaneous SSCD auto-plugging. To our knowledge, the first complete case confirmed on dedicated 3D labyrinthine MRI was reported by Ionescu et al. [17], following an earlier report from the same group describing partial auto-plugging with otolith entrapment [16].

We observed MRI reconstruction images of spontaneous auto-plugging that closely resembled those seen following surgical canal plugging [16,17,30]. Given that patients with SSCD auto-plugging may continue to experience symptoms—particularly autophony—we propose that surgical plugging offers a more complete isolation between the dura and the vestibule. In contrast, auto-plugging appears insufficient to achieve this separation, likely due to the persistence of an “acoustic bridge.” This “incomplete” separation might also account for the presence of abnormally lowered thresholds and increased amplitudes for the cVEMP despite the auto-plugging status, as it can be observed in most patients of our series. Up to this point, we have not found any positive or negative correlation between signs of spontaneous auto-plugging visible on 3D labyrinthine MRI and the presence or absence of lower thresholds for cervical or ocular VEMPs, although a significant SSCD was detected with the HRCT of the temporal bone. Therefore, at this stage, there is no “algorithm” that can predict the possible presence of an early onset of auto-plugging. However, in such cases, we strongly recommend that the HRCT of the temporal bone be carefully reviewed to exclude any other third-window variants in the same ear since in this case clinical TMWA “atypia” may occur. If these have been ruled out, it is important to consider and exclude the possible presence of an early stage of auto-plugging.

### 4.2. Further Pathophysiological Elements

Conceptually, auto-plugging refers to a progressive occlusion of the canal opening by adjacent soft tissues (dura/arachnoid, venous structures, or cicatricial material), thereby partially or completely neutralizing the TMW shunt while introducing new biomechanical constraints on endolymph flow. It is highly probable, considering both the audiological profile and the radiological appearance, that the material responsible for the plug-in phenomenon corresponds to the dura mater and its extensions, rather than bone. This interpretation is supported by imaging data, which consistently show no evidence of abnormal ossification or calcification across this series. The persistence of abnormally low VEMP thresholds suggests that the auto-plugging process does not entirely abolish the dehiscence but rather increases local mechanical resistance. This partial sealing may be sufficient to suppress the Tullio phenomenon and to normalize, at least partially, the audiometric profile. It is therefore plausible that a relatively soft yet elastic dural interface can effectively dampen bidirectional vibratory transmission—both from the intracranial cavity toward the dehiscent canal and from the inner ear toward the dura mater—thereby attenuating the acoustic shunt effect.

Multiple mechanisms could promote not only SSCD but also auto-plugging. First, at the interface, chronic dural or venous contact over a thinned otic capsule—particularly along the middle cranial fossa floor or the superior petrosal sinus—may remodel bone and intermittently seal a dehiscence [31,32]. Second, we hypothesize that co-existing ipsilateral otic-capsule defects (e.g., high riding jugular bulb dehiscence, cochleo-facial dehiscence or CFD) could redistribute pressure and flow, altering micromechanics within the labyrinth and modulating the likelihood that a dehiscent SSC becomes functionally “self-occluded”. We have previously discussed whether the mechanisms proposed for isolated SSCD also apply in cases with multiple dehiscences [25]. In that case, acoustic energy would be expected to be shunted across multiple pathways, producing broader and more diffuse dispersion than with isolated SSCD.

There may also be a connection with mechanical factors such as patients’ nose-blowing habits or anatomical factors like cerebral hemisphere weight or the more apical location of the dehiscence. Systemic factors have also been scrutinized. A Yale cohort did not demonstrate an association between idiopathic intracranial hypertension and radiographic SSCD [33], whereas a subsequent study reported significantly thinner SSC roofs and higher SSCD rates in these patients compared with controls [34]. Taken together, these data suggest that intracranial pressure and related metabolic factors (e.g., obesity) might contribute to auto-plugging in a subset of patients but are unlikely to be necessary or sufficient causes on their own. Clarifying which patients are susceptible—and when—will require adequately powered, prospective imaging-physiology studies with standardized case definitions.

Further, we raise the question of the value of proposing surgical plugging to these patients, who continue to present symptoms despite the radiologic arguments for auto-plugging. The aim would be to close the space responsible for energy leakage to the vestibule and an “acoustic bridge”, in an acoustically tight manner. If plugged surgically, less potential leakage, and therefore potentially more energy available to close the ABG. Most of the patients in our case series had ABG despite self-plugging. Surgical plugging could improve ABG closure, but also autophony and vertigo-like symptoms associated with Tullio. As discussed earlier, the surgical plugging could be made even more complete, sealing the canal all the way up to the ampulla. It has to be noticed that the volume of the endolymphatic system after a plugging procedure is diminished by the surgical procedure and could favor the appearance or persistence of concomitant endolymphatic hydrops [35]. In case of multiple dehiscences (in symptomatic patients), several questions arise: what kind of procedure should be proposed, which dehiscence should be operated on (SSCD, or CFD by round window reinforcement [5]…) and which is the most symptomatic. Proposing a procedure on one of the dehiscences would run the risk of making the other more symptomatic. We have already discussed the need to systematically identify all the otic capsule dehiscences in a patient; we think that in patients with multiple ipsilateral dehiscences, treatment planning should prioritize the dominant shunt and consider endovascular approach when venous–labyrinthine interactions are implicated [25]. Within this present series, there were a few patients in whom the option of surgically completing the auto-plugging process was considered. However, only one patient actually underwent surgery, after all other possible dehiscences on the same symptomatic side had been excluded, and the postoperative result was favorable. Therefore, the principal limitation of this study lies in the fact that only a single patient underwent surgical intervention, which precludes the possibility of drawing statistically meaningful conclusions or establishing robust scientific reference standards at this stage. Nonetheless, we consider it important to emphasize that awareness and consideration of this phenomenon are crucial, particularly in cases demonstrating a positive SSCD on HRCT but exhibiting incomplete clinical manifestations according to the Bárány Society criteria for definitive diagnosis.

## 5. Conclusions

In summary, spontaneous auto-plugging appears to be a plausible phenomenon, increasingly detectable with dedicated MRI techniques, yet likely under-recognized. Potential contributing factors include local dural or venous contact, altered pressure redistribution across multiple labyrinthine windows, and, in selected cases, systemic conditions such as idiopathic intracranial hypertension (IIH). We emphasize the need for prospective, multimodal investigations combining quantitative imaging (CT and high-resolution 3D T2 MRI), vestibular physiology, and longitudinal assessment of symptom trajectories. The development of labyrinth-focused MRI protocols will be critical to capture the onset and progression of auto-plugging and to optimize patient selection for surgical intervention versus conservative management.

## Figures and Tables

**Table 1:** Demographics and instrumental findings.

Patient	Age (Years) Sex	Audiometric Findings Air Bone Gap	Symptoms	VHIT (Gain)	VEMPs Threshold (dB) (Amplitude)
				SSC/HSC/PSC	
1.	68 F	LE: CHL 30 dB (0.25 kHz) 50 dB (0.5 kHz)	Isolated vertigo (sometimes while bending over or blowing nose)	Normal RE: 1/1/1 LE: 0.9/1/1	cVEMPs RE: 95 dB (38.07 µV) LE: 70 dB (550.30 µV) oVEMPs RE:Absent; LE: 95 dB
2.	53 F	LE: mild SNHL (No CHL)	PT, autophony (LE) Dizziness	Normal RE: 1/1/1 LE: 1/1/0.9	cVEMPs RE: 95 dB (428.56 µV) LE: 60 dB (901.89 µV) oVEMPs RE:Absent; LE: 95 dB
3.	77 F	LE: mixed hearing loss; CHL: 40 dB (0.25 kHz)	-Recurrent BPPV despite vestibular rehabilitation	Normal RE: 1/0.8/0.8 LE: 0.9/1/0.9	cVEMPs RE: 95 dB (71.05 µV) LE: 60 dB (948.89 µV) oVEMPs: Not available
4.	85 F	LE: CHL 45 dB (0.25 kHz) 20 dB (0.5 kHz)	-Dizziness. -No auditory symptoms	LE SSC impairment RE: 0.9/0.9/0.7 LE: 0.6/0.9/0.7	cVEMPs RE: 95 dB (35.98 µV) LE: 50 dB (790.74 µV) oVEMPs RE: absent; LE: 95 dB
5.	41 F	LE: CHL 45 dB (0.25 Khz) 20 dB (0.5 kHz)	-Autophony (LE) -Vertigos when sneezing	Normal RE: 0.8/1/1 LE: 0.8/1/1	cVEMPs RE: 50 dB (596.95 µV) LE: 50 dB (585.12 µV) oVEMPs RE: 95 dB; LE: 95 dB
6.	42 M	Bilateral CHL RE: 20 dB (0.25 and 0.5 kHz) LE: 30 dB (0.25 kHz) 15 dB (0.5 kHz)	-Dizziness -Non-PT (bilateral)	Bilateral SSC impairment RE: 0.6/0.9/0.7 LE: 0.6/0.9/0.7	cVEMPs RE: 50 dB (817 µV) LE: 50 dB (728 µV) oVEMPs RE: 70 dB; LE: 60 dB
7.	57 M	SNHL(bilateral) RE: moderate LE: mild	-Autophony (RE) -Tullio -Vertigo at Valsalva (closed glottis) -Dizziness.	Normal RE: 0.9/1/0.8 LE: 1/1/0.8	cVEMPs RE: 60 dB (356 µV) LE: 50 dB (530 µV) oVEMPs RE: 95 dB; LE: 95 dB
8.	41 M	LE: Normal	-Non-PT (LE) (THI 68)	Normal RE: 1/1/1 LE: 1/1/1	cVEMPs RE: 95 dB (150 µV) LE: 95 dB (400 µV) oVEMPs Not available
9.	41 M	LE CHL 30 dB (0.25 and 0.5 kHz)	-Autophony (LE) -PT (LE)	Normal RE:0.8/0.9/0.8 LE:0.9/1/0.9	cVEMPs RE: 70 dB (637.91 µV) LE: 50 dB (1539.67 µV) oVEMPs RE: abs; LE: 90 dB
10.	37 M	LE CHL 40 dB (0.25 kHz) 20 dB (0.5 kHz)	-Ear fullness, PT (LE) -Autophony (LE)	Normal RE: 0.9/0.9/0.9 LE: 0.8/1/1	cVEMPs RE: 95 dB (204.16 µV) LE: 60 dB (755.39 µV) oVEMPs RE: abs; LE: 95 dB
11.	50 F	RE: moderate SNHL LE: CHL 20 dB (0.25 and 0.5 kHz)	-PT (LE)	Normal RE: 1/0.9/1 LE: 0.9/1/1	cVEMPs RE: 95 dB (322.15 µV) LE: 40 dB (1709.61 µV) oVEMPs RE:abs; LE: 60 dB

LE: left ear; RE: right ear; PT: pulsatile tinnitus; BPPV: benign paroxysmal positional vertigo; SNHL: sensori-neural hearing loss; CHL: conductive hearing loss; SSC: superior semi-circular canal; HSC: horizontal semi-circular canal; PSC: posterior semi-circular canal; THI: tinnitus handicap inventory; VHIT: video head impulse test; o and c VEMPs: ocular and cervical vestibular evoked myogenic potentials.

**Table 2:** Radiological data.

Patient	Arguments for HRCT Prescription	Atypia (Suspecting Auto-Plugging)	HRCT	MRI
1.	CHL assessment (on the LE)	Nearly asymptomatic	Unilateral SSCD LE: 4 mm	LE: Partial auto-plugging
2.	Autophony as very invalidating symptom	Mild SNHL, without CHL	Unilateral SSCD LE: 5.2 mm	LE: Partial auto-plugging
3.	Persistent (typical) BPPV after several repositioning maneuvers	Persistent BPPV after repositioning maneuvers	Bilateral SSCD RE: 4 mm LE: 5 mm	LE: Complete auto-plugging
4.	MRI: suspicion of left SSCD CHL assessment (on the LE)	Asymptomatic (including Valsalva) Tumarkin	Bilateral SSCD LE: 4 mm RE: 3 mm (near dehiscence?)	LE: Partial auto-plugging
5. (Figure 2)	LE: CHL and autophony Tinnitus bilateral Vertigo when sneezing	No dizziness, no noise induced vertigo, no PT	Bilateral SSCD RE: 2 mm LE: 5 mm	LE: Partial auto-plugging
6. (Figure 3)	Dizziness Bilateral continuous tinnitus	No vertigo No PT	Bilateral SSCD RE: 7 mm LE: 7 mm	RE: Complete auto-plugging LE: partial auto-plugging
7. (Figure 4)	RE: Autophony Tullio	No CHL No PT	Bilateral SSCD RE: 6.7 mm LE: 1.4 mm	RE: Complete auto-plugging
8.	SSCD suspected on MRI (asked for unilateral no PT)	No CHL No vertigo Tinnitus (No PT)	Bilateral SSCD RE: 6.4 mm LE: 1.9 mm	RE: Complete auto-plugging
9.	LE: CHL	No vestibular symptom	Bilateral SSCD LE: 5.7 mm RE: 6.6 mm	LE: complete auto-plugging RE: partial auto-plugging
10.	LE: CHL Autophony	No pressure or noise induced vertigo	Unilateral SSCD LE: 4.7 mm	LE: complete auto-plugging
11.	SSCD suspected on MRI (unilateral SNHL)	No pressure or noise induced vertigo; No autophony	Bilateral SSCD LE: 4.2 mm RE: 1.5 mm	LE: complete auto-plugging

LE: left ear; RE: right ear; PT: pulsatile tinnitus; CHL: conductive hearing loss; SNHL: sensory-neural hearing loss; BPPV: benign paroxysmal positional vertigo; MRI: magnetic resonance imaging; HRCT: high resolution cranial tomodensitometry; SSCD: superior semicircular canal dehiscence.

## Data Availability

The original contributions presented in this study are included in the article/Supplementary Materials. Further inquiries can be directed to the corresponding authors.

## Supplementary Materials

The following supporting information can be downloaded at https://www.mdpi.com/article/10.3390/jcm14228054/s1, Figure S1: Patient 1. Left SSCD with partial auto-plugging. Left SSCD with partial auto-plugging. (A) HRCT, Pöschl plane; dehiscence measured at 4 mm. (B,C) 3D HR MRI, reconstruction in the plane of the SSC (left ear); Figure S2: Patient 2. Left SSCD with partial auto-plugging. (A) HRCT, Pöschl plane; dehiscence measured at 5.2 mm. (B) fusion between HRCT and 3D T1 MRI. (C) 3D HR MRI, reconstruction in the plane of the SSC; Figure S3: Patient 3. Left SSCD with complete auto-plugging. (A) HRCT, Pöschl plane; dehiscence measured at 4 mm. (B,C) 3D HR MRI, reconstruction in the plane of the SSC; Figure S4: Patient 4. Left SSCD with partial auto-plugging. (A) HRCT, Pöschl plane; dehiscence measured at 4 mm. (B) 3D T2 high-resolution MRI (DRIVen Equilibrium pulse). (C) 3D HR MRI, reconstruction in the plane of the SSC; Figure S5: Patient 8. Right SSCD with complete auto-plugging. (A) HRCT, Pöschl plane; dehiscence measured at 6.4 mm. (B) 3D HR MRI, reconstruction in the plane of the SSC; Figure S6: Patient 9. Left SSCD with complete auto-plugging. (A) HRCT, Pöschl plane; dehiscence measured at 6.6 mm. (B) 3D T2 high-resolution MRI (DRIVen Equilibrium pulse). (C) 3D HR MRI, reconstruction in the plane of the SSC; Figure S7: Patient 10. Left SSCD with complete auto-plugging. (A) HRCT, Pöschl plane; dehiscence measured at 4.7 mm. (B,C) 3D HR MRI, reconstruction in the plane of the SSC; Figure S8: Patient 11. Left SSCD with complete auto-plugging. (A) HRCT, Pöschl plane; dehiscence measured at 4.2 mm. (B) 3D HR MRI, reconstruction in the plane of the SSC.

## Abbreviations

The following abbreviations are used in this manuscript:

ABG	Air bone gap
BPPV	Benign paroxysmal positional vertigo
CFD	Cochleo-facial dehiscence
CHL	Conductive hearing loss
HRCT	High resolution computed tomodensitometry
MRI	Magnetic resonance imagery
PT	Pulsatile tinnitus
SSC	Superior semicircular canal
SSCD	Superior semi-circular canal dehiscence
SNHL	Sensorineural hearing loss
TMW	Third mobile window
VEMP	Vestibular evoked myogenic potentials
VHIT	Video Head Impulse test
VOR	Vestibulo-ocular reflex
