# Supplementary figures and images for: Spontaneous SSCD Auto-Plugging: Clinical, Electrophysiological and Radiological Evidence
Source: J Clin Med. 2025 Nov 13;14(22):8054. doi: 10.3390/jcm14228054 (PMC12653300; doi:10.3390/jcm14228054)

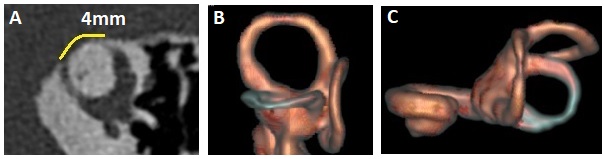

Supplement: Supplementary file 1 [file jcm-14-08054-s001.zip › Figure S1.jpg]

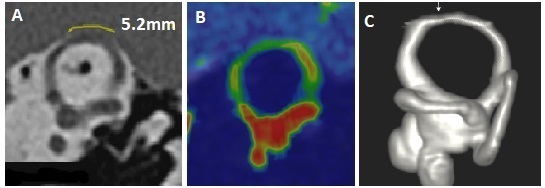

Supplement: Supplementary file 1 [file jcm-14-08054-s001.zip › Figure S2.jpg]

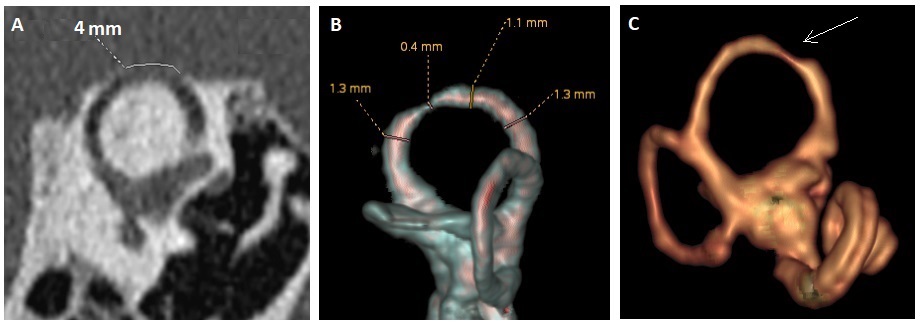

Supplement: Supplementary file 1 [file jcm-14-08054-s001.zip › Figure S3.jpg]

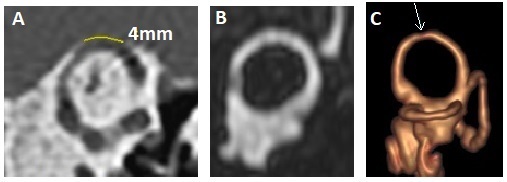

Supplement: Supplementary file 1 [file jcm-14-08054-s001.zip › Figure S4.jpg]

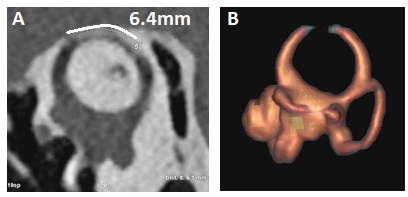

Supplement: Supplementary file 1 [file jcm-14-08054-s001.zip › Figure S5.jpg]

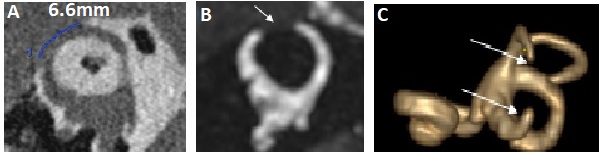

Supplement: Supplementary file 1 [file jcm-14-08054-s001.zip › Figure S6.jpg]

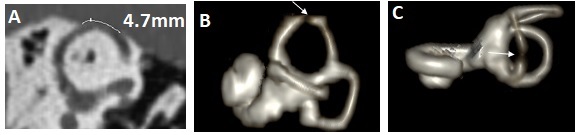

Supplement: Supplementary file 1 [file jcm-14-08054-s001.zip › Figure S7.jpg]

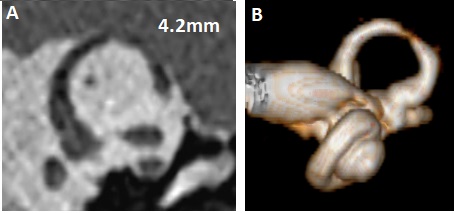

Supplement: Supplementary file 1 [file jcm-14-08054-s001.zip › Figure S8.jpg]
